# Home-based exercise training by using a smartphone app in patients with Parkinson’s disease: a feasibility study

**DOI:** 10.3389/fneur.2023.1205386

**Published:** 2023-06-28

**Authors:** Martina Putzolu, Virginia Manzini, Matteo Gambaro, Carola Cosentino, Gaia Bonassi, Alessandro Botta, Elisa Ravizzotti, Laura Avanzino, Elisa Pelosin, Susanna Mezzarobba

**Affiliations:** ^1^Department of Experimental Medicine, Section of Human Physiology, University of Genoa, Genoa, Italy; ^2^La Colletta Hospital, Azienda Sanitaria Locale 3, Arenzano, Italy; ^3^AISM Rehabilitation Service of Liguria, Genoa, Italy; ^4^Department of Neuroscience, Rehabilitation, Ophthalmology, Genetics, Maternal and Child Health, University of Genoa, Genoa, Italy; ^5^IRCCS Ospedale Policlinico San Martino, IRCCS, Genoa, Italy

**Keywords:** Parkinson disease, exercise, gait, mobile apps, home-based training

## Abstract

**Background:**

Parkinson’s disease (PD) patients experience deterioration in mobility with consequent inactivity and worsened health and social status. Physical activity and physiotherapy can improve motor impairments, but several barriers dishearten PD patients to exercise regularly. Home-based approaches (e.g., via mobile apps) and remote monitoring, could help in facing this issue.

**Objective:**

This study aimed at testing the feasibility, usability and training effects of a home-based exercise program using a customized version of Parkinson Rehab® application.

**Methods:**

Twenty PD subjects participated in a two-month minimally supervised home-based training. Daily session consisted in performing PD-specific exercises plus a walking training. We measured: (i) feasibility (training adherence), usability and satisfaction (via an online survey); (ii) safety; (iii) training effects on PD severity, mobility, cognition, and mood. Evaluations were performed at: baseline, after 1-month of training, at the end of training (T2), and at 1-month follow-up (T3).

**Results:**

Eighteen out of twenty participants completed the study without important adverse events. Participants’ adherence was 91% ± 11.8 for exercise and 105.9% ± 30.6 for walking training. Usability and satisfaction survey scored 70.9 ± 7.7 out of 80. Improvements in PD severity, mobility and cognition were found at T2 and maintained at follow-up.

**Conclusion:**

The home-based training was feasible, safe and seems to positively act on PD-related symptoms, mobility, and cognition in patients with mild to moderate stage of PD disease. Additionally, the results suggest that the use of a mobile app might increase the amount of daily physical activity in our study population. Remote monitoring and tailored exercise programs appear to be key elements for promoting exercise. Future studies in a large cohort of PD participants at different stages of disease are needed to confirm these findings.

## 1. Introduction

Parkinson’s disease (PD) is a neurodegenerative disease that imposes a social and economic burden all over the world with an increasing demand for health care, including pharmacological and surgical treatments, physiotherapy, psychological, and social support services (1). Approximately 0.3% of the general population has PD, rising 1% among people over the age of 60 years (2) with the prevalence increasing as the population ages over 70 (3).

PD is characterized by variable patterns of motor and non-motor symptoms. Indeed, along with motor symptoms such as tremor, bradykinesia, rigidity, postural instability and gait disorders, most patients manifest non-motor symptoms that include, but are not limited to, cognitive impairment, depression, anxiety, pain, fatigue, and sleep disorders (4). Even with an optimal medical management, individual with PD manifest a progressive deterioration in mobility, independence in daily activities and increased risk of falls resulting in a reduced quality of life (QoL). Moreover, as the disease progresses, inactivity become prominent in PD, further deteriorating the health status and social life of patients.

Physical activity and physiotherapy are considered viable adjuncts to pharmacological treatment in improving motor impairments, mobility, and independence in daily life activities, with a positive impact on nonmotor symptoms in PD (4–9). Additionally, recent evidence on animal models and in humans has shown that exercise training may prevent neurodegeneration, regulate neurotrophic factors, and enhance neuroplasticity, suggesting a disease-modifying effect in PD (7). Nevertheless, data suggest that benefits induced by physical activity and physiotherapy programs are not retained over time, especially when training is not performed regularly (6, 10).

In this context, several disease-specific barriers have been recognized to discourage individuals with PD to exercise regularly: lack of time (especially in the early phase), paucity of social support or public transportation, financial sustainability, but also fear of falling, lack of motivation, solitude, or isolation (11). Therefore, to promote long-term adherence to exercise, to encourage an active lifestyle, and to foster pro-active engagement, new solutions need to be implemented (12–14).

In this regard, recent studies showed how a personalized home-based exercise program, with remote monitoring may represent an effective way to promote adherence to physical activity and physiotherapy in individuals with PD (15–17). Furthermore, the growing innovation in health technologies, such as smartphone apps, could pave the way to develop innovative interventions.

Today, only few studies evaluated the efficacy of self-management in PD using mobile applications (16), but there is already evidence of improved walking and mobility, as well as apps usability and applicability (18). However, more research is needed to test the feasibility, the safety, and the efficacy of exercise training delivered via mobile apps and to verify whether this approach could be useful to promote regular engagement in exercise program and physical activity in individuals with PD.

In this context, we designed a feasibility study to test the effects of a minimally supervised home-based exercise program based on a customized version of Parkinson Rehab® app in patients with PD. Specifically, we assessed the feasibility, usability, satisfaction (aim 1), and the safety (aim 2) of a 2-month training performed at participants’ home, and we evaluated training-induced changes on disease severity, mobility, cognition, and mood (aim 3).

## 2. Materials and methods

### 2.1. Participants

Patients with PD were invited to participate in a prospective, open-label, single-arm, single-center feasibility study. Participants were consecutively recruited from the outpatients of the Movement Disorders clinic of the tertiary hospital “IRCCS Policlinico San Martino,” Genova.

The inclusion criteria were as follows: (i) diagnosed with idiopathic PD according to United Kingdom Parkinson’s Disease Society Brain Bank criteria (19); (ii) Hoehn and Yahr stage (H&Y) ≤ 3 (20); (iii) able to walk independently; (iv) stable on antiparkinsonian medication (to maximize patients’ homogeneity) for the past month.

Exclusion criteria were (i) other neurological conditions except PD; (ii) severe cognitive impairment [Montreal Cognitive Assessment score ≤ 17 (21)]; (iii) other comorbidities that would interfere with the protocol adherence (e.g., orthopedic injuries, severe pain, osteoporosis, atrial fibrillation); (iv) visual or acoustic deficits that would restrict the app use; (v) falls episodes that led to emergency care or hospitalization, or > 4 falls within the past year (22).

The study was conducted according to the principles of the Declaration of Helsinki and was approved by the local ethical committee (Comitato Etico Regione Liguria prot. n.771/2021). All participants gave their informed written consent prior to participation.

### 2.2. Exercise intervention

Participants were invited to perform an eight-weeks home-based exercise training, with a minimal supervision, using a customized version of Parkinson Rehab® application, downloaded on their phone. The custom-built app was structured into three main sections: “user,” containing some demographic and clinical information (section “Introduction”), “training” including the exercise program assigned to each participant (section “Materials and methods”), and “steps” collecting and reporting the number of daily steps (section “Results”).

**Figure 1:**
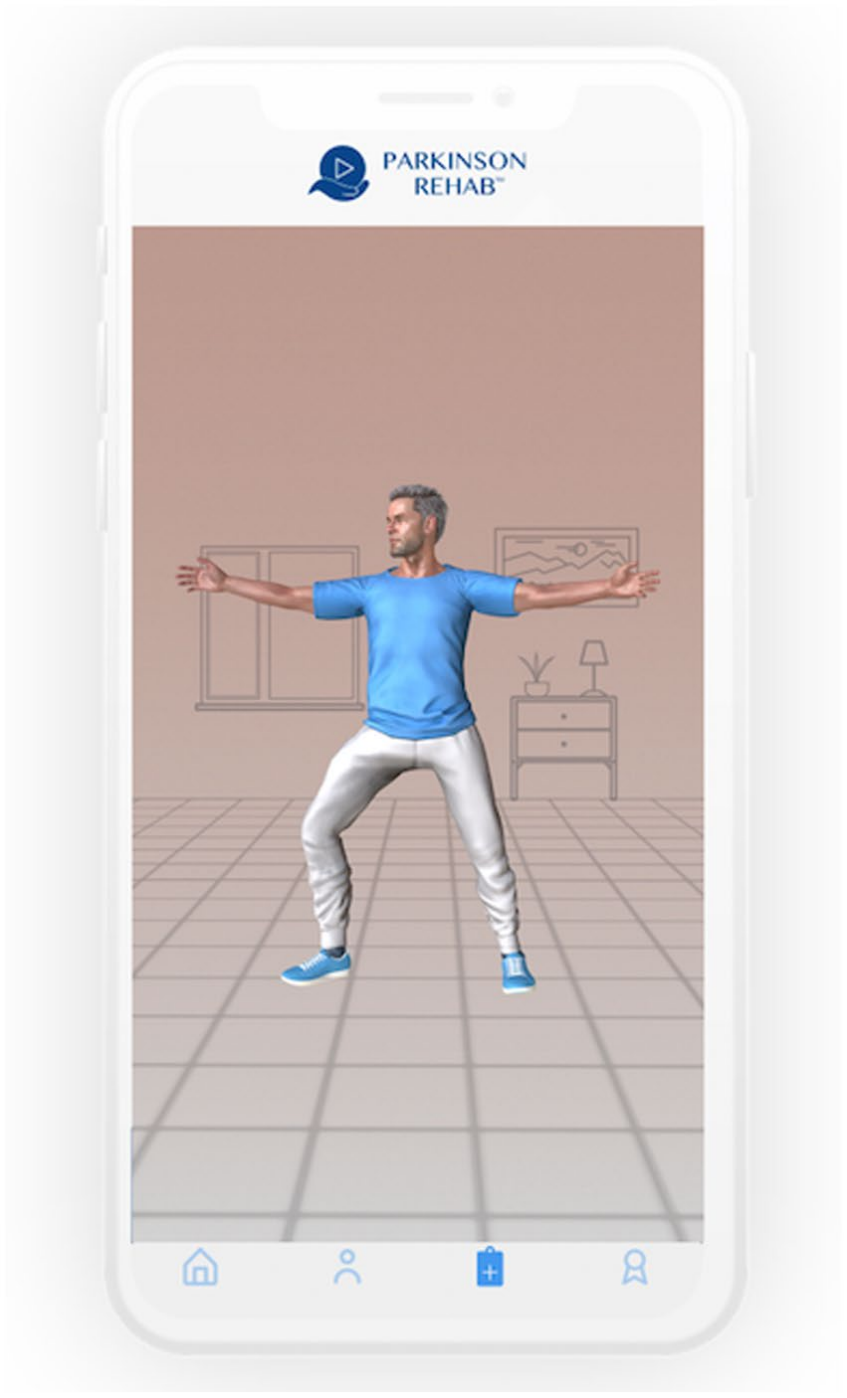
Graphical example of an exercise using the mobile app.

The training program was created with specific exercises for PD-related motor symptoms. The exercises, designed by physiotherapists expert in PD, targeted on postural control, gait, dual tasking, posture, rigidity, bradykinesia, and were proposed in different positions (i.e., laying, sitting, and standing). In all, 42 exercises-videos (performed by an avatar, Figure 1) were included in the app library Supplementary materials (DocS3). As general principle, the training was designed with an increased difficulty of the exercises (i.e., increase in repetitions, sets and in velocity) and with the transition from simpler movements to higher-level activities with more complex motor sequences. To improve customization of the exercise program, the training was initially set based on the participant’s level of function at the baseline, and then revised bi-weekly. Precisely, after baseline evaluation and before starting the training at their home, each patient received a two-week tailored program including: the type, the sets, and the repetitions of the exercises they had to perform daily. Thereafter, participants were phone-called by a physiotherapist to upgrade the exercise program bi-weekly. This approach attempted to provide a training that became more challenging over time, by promoting an active participant’s engagement based on the principles of shaping rehabilitation (23).

To enhance potential benefits of the training, PD participants were instructed to watch each exercise carefully once before starting the training, and to learn the assigned exercises at their best. This instruction was given to exploit action observation training principles (24–26) and to facilitate motor learning processes.

All participants were encouraged to train 7 days a week. The daily program assigned could be completed in one session or split into two parts, depending on each participants’ preference. Duration of each training session was about 30–40 min.

The progression of the exercises was arranged following a pre-determined checklist that analyzed participants’ experience of the previous 2 weeks. Precisely, we asked to participants: (i) feasibility of each exercise and of the entire daily program; (ii) excessive tiredness or weakness during or after the training; (iii) occurrence of any episode of imbalance; (iv) need of a stable support while performing an exercise; (iv) clarity and appreciation of the exercises.

Moreover, to enhance the amount of physical activity patients were invited to follow an incremental walking program. The number of steps x day was set at the baseline visit, based on self-reported average of step taken x day by each participant, and it was increased every two weeks (~ +500 to +1,500 steps). Participants were required to reach the assigned steps 7 days a week and were encouraged to walk even more without any restrictions. The number of steps was collected using the data obtained from the pedometer built in Parkinson Rehab® application. To ensure the most accurate step recording, all participants were instructed to carry their phones with them (e.g., in the pocket or in a waist bag) throughout the day.

Physiotherapists, involved in the project, installed the customized version of Parkinson Rehab® on each participants’ smartphone and trained them how to use the application, before starting the home-based exercise program.

Finally, to promote self-monitoring, patients were required to complete a logbook every day by inserting the number of complete exercise sessions and to note any problems (e.g., forgetting the phone or phone switched off) or to contact the researcher involved in the study in case of any issues.

### 2.3. Outcomes

#### 2.3.1. Aim 1: feasibility, usability, and satisfaction

Feasibility was measured as training program adherence, calculated as: (i) the number of participants who withdrew from the study; (ii) percentage of logbook filling (ratio of total number of days filled out to total number of days of training); (iii) the percentage of the completed training sessions along the entire study (i.e., two months); (iv) the number of exercise sessions completed per month; (v) the ratio of total number of steps performed to total number of steps assigned (from week-1 to week 8) and expressed as a percentage; (vi) the number of daily steps completed per month; (vii) the number of daily steps completed in the first and the last two weeks.

Finally, app usability and participants’ satisfaction were evaluated with an anonymous online survey at the end of the study. Briefly, it consisted of 8 closed questions, scored with a numerical rating scale, and of 2 open-ended questions. Full details are reported in Supplementary materials (DocS1).

#### 2.3.2. Aim 2: safety

Safety was evaluated by recording possible exercise-related adverse events such as falls or near-falls, pain, muscular sprain/strains, fatigue, dizziness, vertigo, or hypotension episodes. The adverse event monitoring was performed every 2 weeks by researchers involved in the training via a phone interview. Detailed information was collected in case any event occurred. Moreover, before starting the training, participants were warned to contact the researchers promptly in case of any issues or questions.

#### 2.3.3. Aim 3: training-induced changes on PD symptoms

Training-induced changes on PD-related symptoms were evaluated using clinical scales or questionnaires. Disease severity was measured using the Movement Disorder Society-Unified Parkinson’s Disease Rating Scale (MDS-UPDRS) (27). Functional mobility was tested with the Short Physical Performance Battery (SPPB) (28) and the Four-Square Step Test (FSST) (29). Balance with the Mini Balance Evaluation System Test (Mini-BESTest) (30), the presence and the severity of freezing of gait (FOG) was assessed using the New Freezing of Gait Questionnaire (NFOGQ) (31). Cognitive and mood status were assessed using the Parkinson Disease-Cognitive Rating Scale (PD-CRS) (27), the Hamilton Anxiety Rating Scale (HAM-A) (32), and the Hamilton Depression Rating Scale (HAM-D) (33).

### 2.4. Procedures

#### 2.4.1. Aim 1

Feasibility outcomes measures were assessed after 1-month of training (T1) and at the end of training (T2), evaluating data collected from the logbook and from the pedometer built in the app. Usability and satisfaction were evaluated at the end of the study by the online survey Supplementary materials (DocS1).

#### 2.4.2. Aim 2

Safety outcomes measures were collected throughout the trial, from baseline to the 1-month follow-up evaluation (T3) in bi-weekly phone interviews.

#### 2.4.3. Aim 3

The clinical outcome measures were assessed at: baseline (T0), within 1-week before training; mid-treatment (T1); end of the training (T2), within 1-week after the training was stopped; and 1-month follow-up (T3). Outcomes were collected about 60 min after oral intake of levodopa (ON-medication phase) and each session lasted about 90 min.

**Figure 2:**
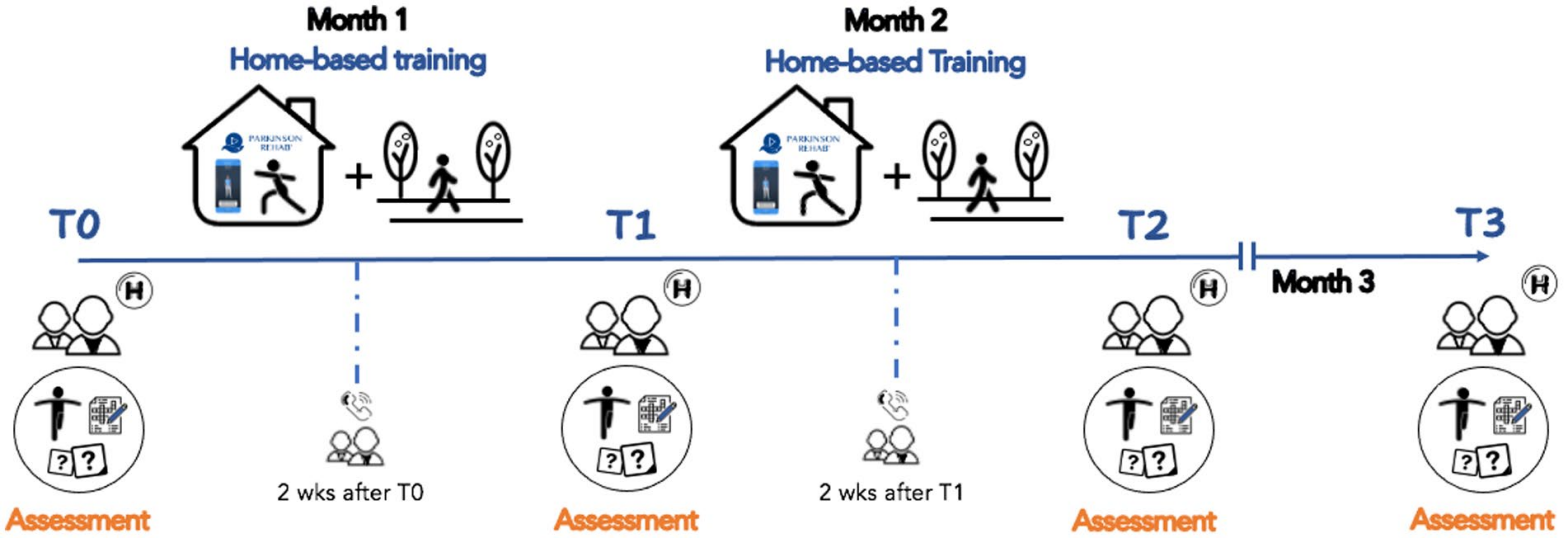
Project timeline. Patients underwent an eight-weeks home-based rehabilitation training with the support of the Parkinson Rehab® application downloaded on their personal smartphones (T0-T2). They participated into 4 evaluations with clinical, cognitive, mood, and motor tests: baseline (T0), one month after the training was started (T1), within 3-days after the end of the training (T2), and at 1-month follow-up (T3). Two weeks after T0 and T1, participants were contacted by phone to provide new exercises and a new number of steps to be taken daily.

A neurologist, specialized in movement disorders, performed the clinical assessment of participants, and researchers (i.e., physiotherapists) involved in the study performed the functional mobility and physical activity evaluations. Timeline of our study is shown in Figure 2.

### 2.5. Statistical analysis

Descriptive statistics (i.e., mean, standard deviation [SD], percentage [%]) were used to report demographical, clinical characteristics of PD participants and participants’ adherence. The normality of the data collected was tested using the Shapiro–Wilk Test.

To detect any change on training program’s adherence between T1 (i.e., mid-training) and T2 (i.e., end of the training) evaluations, a paired *t*-test was used since data were normally distributed.

To be precise, the number of completed exercise sessions per day, and the daily steps performed per day, were inserted in the statistical analysis. In addition, to evaluate whether the number of daily steps performed by participants significantly increased overtime, the number of steps executed the first (week 1 and 2) and the last two weeks (week 7 and 8) were compared using the Wilcoxon signed rank test since they were not normally distributed.

Data obtained from the online survey were analyzed using descriptive statistics (i.e., mean, SD and %) for the first 8 closed questions, judged with a numerical rating scale (0 = not at all, 10 = extremely). Any score < 6 was considered as negative. Results from open-ended questions were categorized based on participants’ answers and reported in Supplementary materials (Doc2).

To assess training-induced changes over time, parametric (one-way Repeated Measure Analysis of Variance, RM-ANOVA) or non-parametric (Friedmann test) analyses were used to compare the clinical data at each evaluation session (T0, T1, T2, T3).

Statistical analysis was performed using Statistical Package for Social Science (SPSS® v.23 for Windows, IBM) and the level of significance was set at *p* < 0.05.

## 3. Results

Among 33 PD patients who were invited to participate, 20 met the inclusion criteria and were inserted in the study. Eighteen PD participants (mean age ± SD: 66.9 ± 7.7 years, 3 females) completed the study and were included in the analysis. Detail of demographic and clinical characteristics of PD participants at baseline are described in Table 1. Results for feasibility and for signal of efficacy on PD symptoms are reported in Table 2.

**Table 1:** Demographic and clinical characteristics of participants at baseline.

Age (years)	66.89 ± 7.65
Sex (% F)	17%
Education (years)	13.83 ± 3.15
Disease duration (years)	7.39 ± 3.97
Falls in the last 6 months (n°)	1.33 ± 2.45
FOG + (n°)	11 PD
FOG - (n°)	7 PD
NFOGQ (score)	12.73 ± 6.54
MDS-UPDRS (total score)	39.94 ± 16.52
MDS-UPDRS part III (score)	22.89 ± 11.45
FSST (sec)	10.83 ± 3.37
SPPB (score)	10.56 ± 1.34
Mini-BESTest (score)	20.61 ± 3.93
PD-CRS (score)	93.88 ± 12.56
HAM-A (score)	10.33 ± 5.95
HAM-D (score)	9.39 ± 6.39

Values are presented as mean ± standard deviation. F, Female; n, number; FOG, Freezing of Gait; NFOGQ, New Freezing of Gait Questionnaire; MDS-UPDRS, Movement Disorder Society–Unified Parkinson’s Disease Rating Scale; FSST, Four Square Step Test; sec, seconds; SPPB, Short Physical Performance Battery; Mini-BESTest, Mini Balance Evaluation Systems Test; PD-CRS, Parkinson’s Disease-Cognitive Rating Scale; HAM, Hamilton Anxiety Rating Scale; HAM-D, Hamilton Depression Rating Scale.

**Table 2:** Results summary regarding feasibility and effects on PD symptoms.

Feasibility	T1	T2	*p*-value
Exercise training adherence (%)	93.07 ± 13.27	88.87 ± 13.05	0.16
2-month Exercise training adherence (%)	–	90.97 ± 11.75	–
Walking program adherence (%)	110.37 ± 34.46	102.78 ± 33.85	0.32
2-month Walking program adherence (%)	–	105.87 ± 30.59	–
	Week1&2	Week7&8	*p*-value
Daily steps (*n*)	6499.65 ± 2618.46	7466.01 ± 3116.59	<0.0001

Effects on PD symptoms	T0	T1	T2	T3	*p*-value (TIME)
MDS-UPDRS [total score]	39.94 ± 16.52	33.72 ± 15.54	32.78 ± 15.09	29.94 ± 14.21	*F*_(1.87, 31.71)_ = 5.25, *p =* 0.01
*(post-hoc)*		(*p* = 0.01)	(*p* = 0.03)	(*p* = 0.002)	
MDS-UPDRS part III [score]	22.89 ± 11.45	18.39 ± 11.12	17.56 ± 9.99	15.67 ± 9.51	*F*_(1.73, 29.45)_ = 4.92, *p* = 0.02
*(post-hoc)*		(*p* = 0.01)	(*p* = 0.03)	(*p* = 0.002)	
NFOGQ [score]	11.67 ± 7.24	10.50 ± 6.88	11.17 ± 7.02	10.58 ± 7.72	*χ*^2^(3) = 2.72, *p* = 0.44
	*(post-hoc)*	(*p* = 0.33)	(*p* = 0.64)	(*p* = 0.50)	
Mini-BESTest [score]	20.61 ± 3.93	23.06 ± 3.56	22.61 ± 4.16	22.83 ± 4.49	*F*_(3,51)_ = 5.31, *p* = 0.003
*(post-hoc)*		(*p* = 0.002)	(*p* = 0.02)	(*p* = 0.02)	
SPPB [score]	10.56 ± 1.34	11.00 ± 1.03	11.22 ± 0.94	11.33 ± 0.97	*χ*^2^(3) = 14.56, *p* = 0.002
*(post-hoc)*		(*p* = 0.11)	(*p* = 0.01)	(*p* = 0.01)	
FSST [sec]	10.59 ± 3.31	10.17 ± 3.34	9.15 ± 3.00	9.15 ± 3.02	*χ*^2^(3) = 15.75, *p* = 0.001
*(post-hoc)*		(*p* = 0.33)	(*p* = 0.01)	(*p* = 0.003)	
PD-CRS [score]	93.88 ± 12.56	99.10 ± 11.04	102.71 ± 12.08	103.13 ± 14.53	*χ*^2^(3) = 21.6, *p* < 0.001
*(post-hoc)*		(*p* = 0.01)	(*p* = 0.001)	(*p* = 0.001)	
HAM-A [score]	10.33 ± 5.95	9.83 ± 5.25	9.11 ± 5.01	7.89 ± 4.42	*F*_(2.01, 34.23)_ = 1.16, *p =* 0.33
*(post-hoc)*		(*p* = 0.80)	(*p* = 0.40)	(*p* = 0.09)	
HAM-D [score]	9.39 ± 6.39	9.78 ± 6.86	8.33 ± 5.57	7.83 ± 6.11	*F*_(3,51)_ = 1.48, *p* = 0.23
*(post-hoc)*		(*p* = 0.71)	(*p* = 0.28)	(*p* = 0.25)	

Values are presented as mean ± standard deviation. n, number; PD, Parkinson’s Disease; MDS-UPDRS, Movement Disorder Society–Unified Parkinson’s Disease Rating Scale; NFOGQ, New Freezing of Gait Questionnaire; Mini-BESTest, Mini Balance Evaluation Systems Test; SPPB, Short Physical Performance Battery; FSST, Four Square Step Test; sec, seconds; PD-CRS, Parkinson’s Disease-Cognitive Rating Scale; HAM-A, Hamilton Anxiety Rating Scale; HAM-D, Hamilton Depression Rating Scale.

### 3.1. Feasibility

Drop-out rate was 10%, indeed 18 participants completed the entire protocol, and two patients withdrew for personal reasons unrelated to the nature of the study protocol. The logbook filling was more than satisfactory (94%), indeed only one participant did not complete the diary.

Results for the exercise training revealed a high rate of participants’ adherence to the program assigned, with a 91% (± 11.8 SD) of completed sessions: 12 participants were above 90%, four between 75 and 90%, and one slightly above 50%. Statistical analysis did not show significant difference on exercise sessions completed at T1 (mid-training) and T2 (end of the program) evaluations (mean ± SD: 93.1 ± 13.3% at T1; 88.9 ± 13.1% at T2, *p* = 0.16).

**Figure 3:**
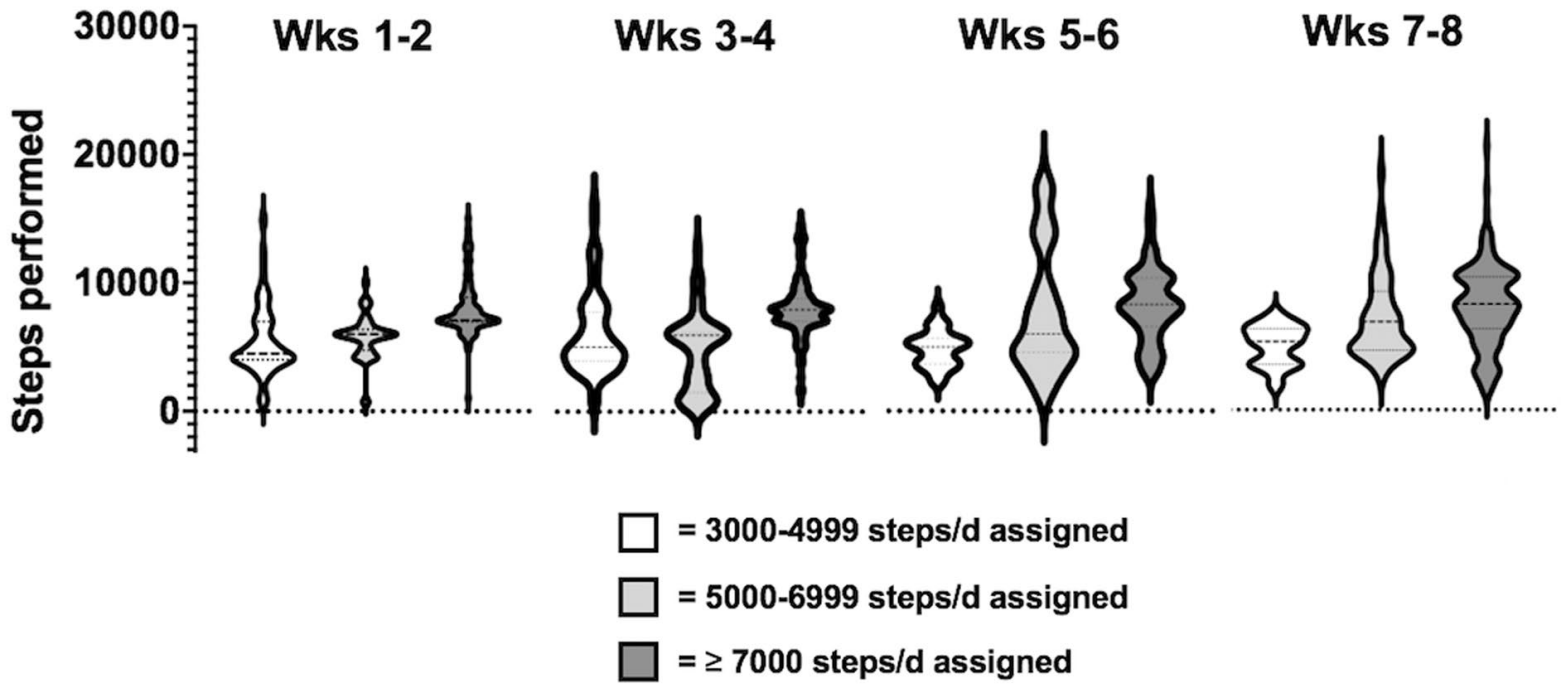
Distribution of bi-weekly steps performed by participants throughout the total training period, clustered based on steps assigned x day (3000-4999 steps, 5000-6999 steps, ≥ 7000 steps).

Regarding the amount of physical activity, the daily steps assigned to the participants ranged from 3,500 to 8,000 steps in the first two weeks, up to a range of 5,000 to 10,000 steps in the last two weeks. Data collection for daily steps were satisfactory: data were complete (i.e., 56 days) in 16 participants (70%), 1-day data (i.e., 55 out of 56 days collected) in 5 participants (25%) and 2-day data, in 1 patient, were excluded from the analysis, because participants declared they did not carry the phone with them during the day or because data by the app failed. Results revealed that the adherence to the assigned daily steps was on average of 105.9% (±30.6 SD) over the entire period of training. The total number of steps performed by each participant ranged from 232.554 to 566.065, with 11 patients exceeding the assigned target (range: from +0.1% to +104.7%) and 6 patients failing to achieve the goal (range: from-1% to-27.8%). Distribution of bi-weekly steps performed by participants over the two-month, clustered based on steps assigned (3,000–4,999 steps, 5,000–6,999, ≥ 7,000), is shown in Figure 3.

Statistical analysis on participants’ adherence to the daily steps program assigned, at T1 and T2, was not significant (mean ± SD: 110.4 ± 34.5% at T1; 102.8 ± 33.9% at T2, *p* = 0.32). In addition, a significant increase of daily steps performed by participants the first (week 1 and 2; mean ± SD: 6499.7 ± 2618.5) and the last two weeks (week 7 and 8; mean ± SD: 7466 ± 3116.6) was found (*Z* = −4.3 *p* < 0.0001).

Regarding Parkinson Rehab App® usability and the overall satisfaction with training protocol, the mean total score of the 8 closed questions, according to a 10-point numerical rating scale, was of 70.9 (± 7.7 SD) out of a maximum of 80.

The highest score was observed for “Were the explanations about using the application clear?” (Question n. 4, mean score 9.4 ± 0.9 SD), followed by “Do you think this application is structured in a functional and practical way” (Question n. 3, mean score 9.2 ± 1.1 SD). Based on the cut-off <6 established to detect issues or negative impressions from participants, a score of 2 was given by only one participant to the question “Do you think this application is easy to use?.” Plus, two participants assigned a score of 5 for the question “Would you recommend this application to other people with Parkinson’s disease?”

Based on the responses obtained from participants regarding the best and the worst aspects of using the app (question n.9 and n.10, respectively) the answers were divided into three categories as follows: “Training” (i.e., the type of exercises and training program proposed), “User-friendly,” and “Perceived benefits” for question n.9; “Technological issues” (e.g., problem with internet connection), “Time commitment,” and “No perceived benefits,” for question n.10. Also, “Nothing to Report” was used when participants responded that they had nothing to declare.

Concerning the best features of using the app, most of the participants answered “Training,” (*n* = 6) and “Perceived benefits” (*n* = 5), while for the worst aspects, most of PD patients had Nothing to Report (*n* = 10) and 5 reported technological issues, not related to the app. Detailed results are reported in Supplementary materials (DocS2).

### 3.2. Safety

The primary outcome for safety were training program-related adverse events. Any severe adverse event was reported by participants during the entire training program. The phone interview revealed no report of falls or near-falls, muscular sprain/strains and dizziness or vertigo during the app-based exercise training. Only two episodes of weakness after the exercise training were recorded. No patient missed bi-weekly phone calls. In addition, results of the question *“Did you feel safe while exercising with the Parkinson Rehab® application?”* from the online survey showed that participants felt the app safe (mean score 9.1 ± 1.2 SD). Details of individual scores are shown in Supplementary materials (DocS2).

### 3.3. Effects on PD symptoms

Results are reported in Table 2. Overall, statistical analysis indicated significant TIME effects for all the variables measured (*p* always <0.05), except for NFOGQ (*χ*^2^(3) = 2.72, *p* = 0.44), HAM-A (*F*(2.01, 34.23) = 1.16, *p* = 0.33), and HAM-D (*F*(3,51)=1.48, *p* = 0.23).

*Post-hoc* analysis revealed significant improvements at the end of the training (T0 vs. T2) for disease severity (MDS-UPDRS, total score *p* = 0.03; part III, *p* = 0.03), balance, evaluated by Mini-BESTest and FSST, (*p* = 0.02 and *p* = 0.01, respectively) and mobility (SPPB, *p* = 0.01). Also, these improvements were maintained up to the 1-month follow-up evaluation (T0 vs. T3, MDS-UPDRS total score, *p* = 0.002; part III, *p* = 0.002; Mini-BESTest, *p* = 0.02; FSST, *p* = 0.003 and SPPB, *p* = 0.01).

**Figure 4:**
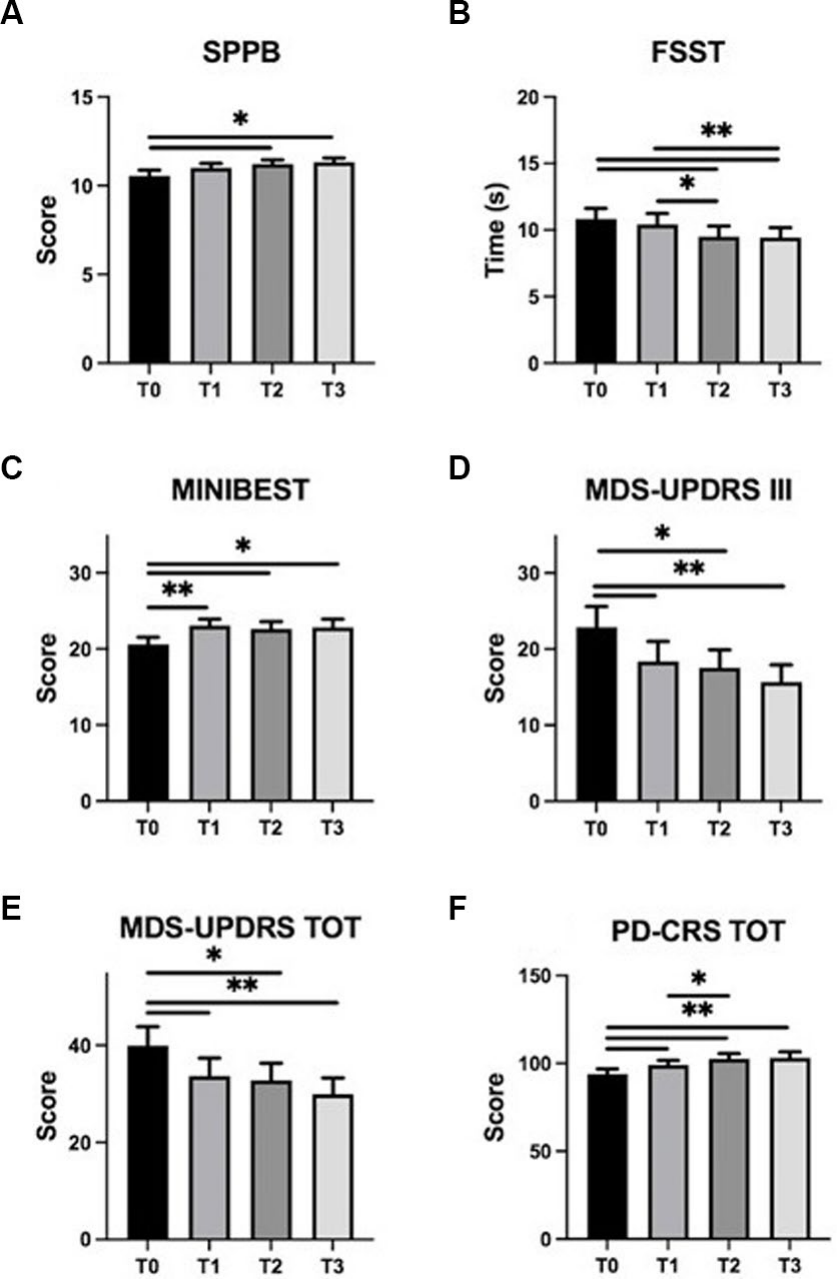
Training-induced changes on PD symptoms. SPPB, FSST, Mini-BESTest, MDS-UPDRS (part III and total), and PD-CRS mean scores (± standard error) are represented at T0 (baseline), T1 (mid-training), T2 (end of the training), and T3 (one month follow up). Panels: **(A)** SPPB, Short Physical Performance Battery; **(B)** FSST, Four Square Step Test; **(C)** MINIBEST–Mini Balance Evaluation Systems Test; **(D)** MDS-UPDRS III, Movement Disorder Society–Unified Parkinson’s Disease Rating Scale part III; **(E)** MDS-UPDRS TOT, Movement Disorder Society–Unified Parkinson’s Disease Rating Scale total score; **(F)** PD-CRS TOT, Parkinson’s Disease-Cognitive Rating Scale total score. Asterisks indicate statistically significant differences emerged at *post hoc* analysis of RM-ANOVA or Friedmann test (∗*p* < 0.05; ∗∗*p* < 0.01).

Interestingly, when potential effects of the exercise program were measured at mid-time training (T1) some significant changes were already observed for disease severity (T0 vs. T1, MDS-UPDRS, total score *p* = 0.01 and part III, *p* = 0.01) and for balance [Mini-BESTest score (*p* = 0.002), but not for FSST (*p* = 0.33) and for SPPB (*p* = 0.11)]. Results related to training-induced changes on PD symptoms are shown in Figure 4.

Regarding cognition and mood status, statistical analysis revealed significant effect of TIME on PD-CRS total score (*χ*^2^(3) = 21.6, *p* < 0.001). *Post-hoc* analysis showed improvements at T1 (*p* = 0.01), T2 (*p* = 0.001) and T3 (*p* = 0.001) evaluations compared to data obtained at baseline (T0).

## 4. Discussion

The objectives of this exploratory study were to test the feasibility, safety, and the possible clinical induced-changes of an app-based training program in patients with PD. Participants were invited to exercise by themselves at their home for 8 weeks using a customized version of Parkinson Rehab® application. In addition, to monitor participants’ adherence and to mitigate the barriers related to unsupervised training (17) (i.e., high rate of drop-out) a minimal remote supervision was provided.

Overall, results showed that a customized training provided by an app may be feasible and safe for individuals in the mild to moderate stage of PD disease. All participants increased their daily physical activity, and improved mobility and balance. Improvements on disease severity, PD motor signs and global cognition were also found.

### 4.1. Feasibility and safety

The primary aims of our study were to evaluate the feasibility, usability, and satisfaction (aim 1) and the safety (aim 2) of a customized training provided through an app specifically designed for targeting PD symptoms. Together, our results showed a high adherence to the exercise training (about 91%) and to the daily-steps program (about 106%) assigned within two months training. The low drop-out rate (10%) and the high level of satisfaction (survey question n°1-mean score: 8.9 /10) express a very good level of engagement to the app-guided training. Moreover, PD participants reported that the app was easy to use, the exercises were clearly explained, and they would recommend its use to others individual with Parkinson’s disease. No severe adverse event (i.e., falls or injuries) were reported during the study period and only two individuals report one episode of weakness after the daily exercise session, suggesting that the training provided through Parkinson Rehab® app was safe. This was also confirmed by the satisfaction survey, where the safety was judged as high by most of the participants (question n°6-mean score 9.1/10).

The idea to promote continuity of care and to improve motor impairments and cognitive decline by using phone or tablet-based apps in neurological diseases has been increasingly investigated in the past decade, and it has received an additional boost from the COVID-19 pandemic (34). In the field of movement disorders, three recent works tested feasibility, usability, and the safety of home-based training via customized apps in patients with PD (18, 35) and Parkinsonism (36). The first work (35) tested, in a pilot randomized controlled trial (RCT), the preliminary acceptability, safety and effectiveness of a mobile health (mHealth)–mediated exercise program in PD. The mHealth program was compared over 1 year, with an exercise program administered without mobile technology (e.g., exercises were provided in a paper format with photos and general instructions). Twenty-six PD participants were allocated in the m-Health protocol and 25 in the control group. Feasibility was measured as the adherence of the program assigned (daily steps and exercises performed). Both interventions were well-tolerated and received favorable satisfaction ratings. The percentage of participants that wanted to continue the exercises program was high in both groups (82% app group, 70% active control group) and the dropout rate was low (11% app group, 16% active control group). The intervention was judged as safe: no difference in the number of adverse events was seen between groups and any serious event occurred.

Thereafter, the same research group (18) tested, in a single-cohort pilot study, the feasibility and the efficacy of an unsupervised exercise home-based training using a commercially available mobile app in individuals with PD. Participants, who had already downloaded the app, were automatically invited to take part of the study. Demographical, clinical and mobility data were collected by the app. Based on this information, a customized training program was assembled via an *ad hoc* algorithm and directly assigned to the participants which were encouraged to train at least 150 min per week. Results revealed that about the 43% (12 out of 28) of the participants averaged more than 150 min of app usage per week and that the remaining 16 averaged from 120 to <90 min per week. Usability was tested using a 5-point Likert questionnaire at the end of the training (12 weeks). Results (19 out 28 respondents) revealed that a video-guided training delivered via mobile app was safe, enjoyable, and appropriate for their level of function for ~70–85%, whereas for ~5% was neutral and for ~5% was not fully satisfied. However, a high dropout rate of participants (40%, 19 over 47) was registered over the course of the study.

More recently, Kim (36) and co-workers designed a prospective, open-label, single-arm pilot study to test the effects of a home-based remotely supervised reinforcing exercise program (8 weeks) with a custom-made app in individuals with PD (n = 13) or atypical parkinsonism (*n* = 8). Each day the app showed the total number of exercises participants had to complete and provided alarms notification to promote participants’ motivation and adherence.

Usability was measured with a self-reported questionnaire and the answers scored using a 7-point Likert-type scale. Overall, the results showed that participants were satisfied (mean score: 5.3/7), perceived as adequate the training assigned (mean score 5.2/7) and they were quite supportive of using the app also in the future (i.e., “intention to use” question, means score 6.1/7). The drop-out rate was quite low (about 14%) and only 1 out of the 3 participants dropped out for reason related to the study (i.e., difficulty in using the app).

Our results, together with previous finding, suggest that home-based exercise trainings provided via apps are safe and feasible, supporting the idea that they may represent a valid option to promote continuity of care in the early to mild phase of PD. However, to contribute to this growing area of research, some aspects are worthy of discussion.

First, results on participants’ adherence to the program are still mixed, with a drop-out rate ranging from 10 to 40%. As previously reported one of the factors that may hinder the long-term adherence to app-based program is the lack of direct contact with clinicians during the entire duration of the training. *Vice-versa* a blended approach, including a remote supervision seems to facilitate engagement and continuity to home-based program provided using mobile or web apps (14, 37). This could explain the low drop-out rate registered in our study (about 10%), that is similar to that reported by Kim et al. (about 14%). Additionally, in accordance with patient monitoring and coaching of the Chronic Care Model (11, 35, 38), we planned periodic phone calls and the compilation of a daily exercise diary. At the end of the study, all participants completed 100% phone counselling and results revealed a high compliance (94%) in daily logbook reporting. This result is consistent with previous work showing that telephone counselling improve adherence to mobile health intervention for self-management of PD (16). However, we did not collect quantitative or qualitative data on phone calls, so it is difficult to know how and how much the phone conversation influenced the active participation to the study.

Second, frequent program adaptation is considered another key element in promoting participants’ active engagement and in reducing the possibility of the adverse events occurring. Indeed, as previously reported (39), PD patients expect to be included in an individualized physiotherapy intervention to achieve greater improvements and to minimize their motor disabilities. Results from our and previous work (36), although preliminary, support this hypothesis showing that supervised management by physiotherapist, expert on PD, may have a greater impact on participants’ adherence and may be superior in developing a more tailored program compared with no supervision or the use of automatic algorithms. In addition, the use of purpose-built apps tailored to meet the specific needs of patients, can assist physiotherapists in effectively supervising and quantifying the daily exercises carried out by patients and in adjusting the training based on their results or perceived barriers. This could help in enhancing participants’ long-term adherence to the training program, thereby promoting long-lasting benefit of exercise and physical activity.

### 4.2. Effects on PD symptoms

The Aim 3 of our study was to assess possible training-induced changes on motor symptoms in PD and to test potential effects on cognitive functions and mood. In line with previous finding (40–42), we found that 8-weeks of training were sufficient to lead to significant improvements on PD motor symptoms, mobility, and balance with a positive impact on global cognition. Plus, results at follow-up showed that these improvements were maintained up to 1-month after the end of training. Conversely, any significant change was detected for mood and anxiety.

Encouraging results of exercise programs via mobile apps on motor and non-motor symptoms in PD have previously reported. The first published pilot RCT (35) aimed at promoting sustained physical activity in people with PD. Participants were invited to perform 5 to 7 exercises for ≥3 days-week and to perform a daily walking program (from 5,000 to 10,000 according to each participant’s activity level at the baseline). Results showed that 12-month individually tailored home exercise and walking program enhanced with mobile technology was comparable to a “standard” training (i.e., without the app) in improving general physical activity, measured as changes in daily steps, and in increasing walking capacity (i.e., increased meters walked during the 6 min walking test). However, sub-analysis results revealed that changes in daily steps and moderate-intensity minutes were clinically meaningful in the “less” active compared to the “more” active group when the app was used. This result was attributed to some additional elements, such as rewards, and notification, which are known to promote adherence and behavioral changes in elderly and healthy adults (43, 44). Despite fairly positive results, it should be taken into account that the accuracy of steps measurement via phone’s built-in sensors, still has limitations because they are estimated. Therefore, these results, as well as ours, must be interpreted with caution.

Later, Landers and collaborators (18) tested the effects of an exercise program, delivered using a customized app, on mobility in patients with PD. Participants were asked to perform a personalized training program for at least 150 min a week, for 12 weeks. The exercises were proposed as video-clip and were divided into three main categories (strengthening, balance, and stretching exercises). The app used the results from the self-report questions and the performance-based measurements to adjust type, length, and intensity of the exercises thanks to a customized algorithm. At the end of the training, results showed significant improvements for lower extremity strength and dynamic balance and mobility (measured using the Timed Up and Go test) in PD participants in the early stage of the disease. Interestingly, any statistically difference between mid-time (8-week) and final (12-week) evaluations for any outcome was found, suggesting that improvements plateaued by the 8-week measurement point. Finally, results from a recent work (36) showed that a training based on a customized mobile app might increase duration and frequency of daily exercises in a small cohort of PD participants (n.13), although neither motor symptoms nor functional mobility were objectively assessed.

Overall, these results suggested that app-based training program may lead not only to health benefits in general, but also may act on PD-related symptoms. This is in line with extensive scientific literature showing that physical activity and physiotherapy program impact on disease progression (7) and improve motor, and non-motor symptoms in PD (8).

In this context, further possible benefits of using mobile apps for providing home-based training may be the possibility to adapt the training program quickly, to couple several exercise modalities easily (45) and to incorporate difference training program (e.g., motor and cognitive). Also, a further advantage of using mobile apps could be identified in the way the exercises are proposed, which is “observation plus imitation” (i.e., action observation therapy, AOT). Indeed, the use of AO, as a modality for delivering the exercises, might have contributed to the observed improvements achieved by participants, by promoting the coupling of the observed actions with internal representations of movements. This neural mirroring process allows the observed action to be simulated in the brain, enabling not only the understanding and comprehension of others’ actions, but also enhancing motor learning and imitation processes. Therefore, patients could have strengthened the formation of motor memories by observing and imitating the exercises, which may have in turn led to further improve motor performance. This is supported by several works (46) showing that AOT, which enable the mirror neurons system, is a valuable strategy to improve motor performance (24), dual tasking and to promote motor learning (23) in PD patients. Furthermore, the requirement to look carefully at the exercises before performing them, drives patients to pay attention to their movements and to use their residual executive resources (47). These modalities (observation, imitation and explicit instruction) allow to reinforce the cortical mechanisms involved in the execution of the movement by activating the volitional-executive motor control system, and thus circumventing the dysfunctional, habitual, sensorimotor basal-ganglia network (47, 48).

Regarding cognition, our results showed a significant increase on PD-CRS score at the end of the training and at one-month follow up. Backwards, no improvement on anxiety and depression was detected. Changes on cognitive ability induced by physical activity (e.g., aerobic training) and physiotherapy (e.g., dual-tasking, virtual reality) has been already reported in PD and the assumed underlying mechanisms may vary depending on the type of training applied (7). Here, we might hypothesize that our training program may have acted also on cognitive functions, such as attention, working memory and spatial ability, because participants were required to closely follow and imitate the exercises seen on the screen, to dual-task and to focus on different elements of the movements (e.g., sequence, speed, amplitude). However, these results should be interpreted with caution, as our training program did not include a section devoted specifically to cognitive function training.

Finally, our results did not show any significant improvement on FOG. This is not surprising because it is known that specific interventions (49, 50) and exercises (51) to overcome freezing episodes are essential to improve FOG symptoms and our training program was not designed to specifically address this problem.

Several limitations of the study deserve attention. First, this is a feasibility study and controlled trials (e.g., RCT) are needed to confirm our results. Second, participants were consecutively enrolled and thus our sample was not gender-balanced, resulting in 15 males and 3 females. Third, our study was based on an 8-week training program, so we did not test participants’ adherence over a longer period of time. Fourth, we scheduled only one and short-term follow-up (one-month), therefore longer-term effects of the training program was not verified. Fifth, we did not monitor the support or the assistance of the caregiver during the training. Sixth, in our study, we did not collect any quantitative measures of gait, but we solely recorded the amount of walking performed daily by each participant. Finally, caution is required in interpreting data collected through phone built-in sensors (e.g., number of steps), as they are not comparable with gold-standard measurements and they may differ depending on the phone’s hardware (e.g., motion sensors quality).

## 5. Conclusions and future perspective

Our finding suggests that a multimodal, partially supervised, home-based training with a customized app is feasible, safe and seems to improve physical activity, mobility, and PD related symptoms. This type of intervention seems ideal for promoting self-management and continuity of care and for changing motor habits. However, relevant barriers hampering a continuous app usage, such as technology-related issues or lack of compliance or adherence, are yet to be fully investigated. Conversely, remote monitoring by clinicians appears to be a key element for promoting adherence and to adjust training program according to participants’ need over time, toward a precision medicine approach.

A further step for the next-generation exercises apps would be to integrate clinical and rehabilitative intelligence assessment systems, by using modern wearable devices and new Machine Learning (ML) algorithms. This would enable to gather additional clinical data, such as those related to disease severity and its progressions, and to perform clinical assessments, for example those related to mobility, thus helping clinicians to provide optimal care even at patients’ home (52, 53). Increasing and improving the quality of clinical data collection would help clinicians to provide optimal care even at patients’ home and it might promote the creation of extensive datasets useful for the growth of new patient-centered model of care (54).

## Data availability statement

The raw data supporting the conclusions of this article will be made available by the authors, without undue reservation.

## Ethics statement

The studies involving human participants were reviewed and approved by Comitato Etico Regione Liguria. The patients/participants provided their written informed consent to participate in this study.

## Author contributions

MP, LA, EP, and SM: conceptualization. MP, CC, GB, and SM: formal analysis. VM, MG, AB, and ER: investigation. LA, EP, and SM: project administration. EP: supervision. MP, EP, and SM: writing – original draft. MP, CC, GB, LA, EP, and SM: writing – review and editing. All authors have read and agreed to the published version of the manuscript.

## Funding

This work was partially supported by the Ministry of Health, project NET-2016-02361805, “E-Action” 02361805–4 and by #NEXTGENERATIONEU (NGEU) and funded by the Ministry of University and Research (MUR), National Recovery and Resilience Plan (NRRP), project MNESYS (PE0000006) – A Multiscale integrated approach to the study of the nervous system in health and disease (DN. 1553 11.10.2022).

## Conflict of interest

The authors declare that the research was conducted in the absence of any commercial or financial relationships that could be construed as a potential conflict of interest.

## Publisher’s note

All claims expressed in this article are solely those of the authors and do not necessarily represent those of their affiliated organizations, or those of the publisher, the editors and the reviewers. Any product that may be evaluated in this article, or claim that may be made by its manufacturer, is not guaranteed or endorsed by the publisher.
