# Supplementary material for: Home-based exercise training by using a smartphone app in patients with Parkinson’s disease: a feasibility study
Source: Front Neurol. 2023 Jun 28;14:1205386. doi: 10.3389/fneur.2023.1205386 (PMC10338039; doi:10.3389/fneur.2023.1205386)
Supplement: Supplementary file 1 [file Table_1.DOCX]

Supplementary Material

**Home-based exercise training by using a smartphone app in patients with Parkinson’s Disease: a feasibility study.**

**Martina Putzolu**1, Virginia Manzini2, Matteo Gambaro3, Carola Cosentino4, Gaia Bonassi4, Alessandro Botta5, Elisa Ravizzotti4, Laura Avanzino1,5, **Elisa Pelosin4,5*** and Susanna Mezzarobba4,5

*** Correspondence:** Corresponding Author: elisa.pelosin@unige.it

# Supplementary File

| **Survey evaluating the usability of Parkinson Rehab® app and the general experience with the motor training protocol** |
| --- |
| 1. How satisfied are you in general with your experience with this training program? (0-10)  2. Do you think this application is easy to use? (0-10)  3. Do you think this application is structured in a functional and practical way? (0-10)  4. Were the explanations on using the application clear? (0-10)  5. Are the movements made by the avatar easy to understand? (0-10)  6. Did you feel safe while exercising with the Parkinson Rehab® application? (0-10)  7. Do you think that using the application was a valid tool for doing exercises at home? (0-10)  8. Would you recommend this application to other people with Parkinson's disease? (0-10)  9. What did you like most about your experience using the Parkinson Rehab® application? (Open answer)  10. What did you like least about your experience using the Parkinson Rehab® app? (Open answer) |
| **Notes.** Questions 1-8 use a 10-point numerical rating scale ranging from 0 = "not at all" to 10 = "extremely" |
