# Supplementary material for: Home-based exercise training by using a smartphone app in patients with Parkinson’s disease: a feasibility study
Source: Front Neurol. 2023 Jun 28;14:1205386. doi: 10.3389/fneur.2023.1205386 (PMC10338039; doi:10.3389/fneur.2023.1205386)
Supplement: Supplementary file 2 [file Table_2.docx]

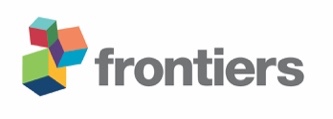
Supplementary Material

**Home-based exercise training by using a smartphone app in patients with Parkinson’s Disease: a feasibility study.**

**Martina Putzolu**1, Virginia Manzini2, Matteo Gambaro3, Carola Cosentino4, Gaia Bonassi4, Alessandro Botta5, Elisa Ravizzotti4, Laura Avanzino1,5, **Elisa Pelosin4,5*** and Susanna Mezzarobba4,5

*** Correspondence:** Corresponding Author: elisa.pelosin@unige.it

# 2 Supplementary Table

**Table 2.** Results of the survey evaluating app usability and general experience with the training protocol. Questions 1-8 were assessed with a 10-point numerical rating scale (0 = "not at all" - 10 = "extremely"). Positive aspects (question 9) were categorized in " Training ", " User-friendly", and " Perceived benefits "; negative aspects (question 10) were classified in "Technological issues", "Time commitment", and " No perceived benefits”.

Column "Total score" reports: (i) the sum of scores obtained from questions 1-8, (ii) a percentage score (in brackets) in which a score of 80 corresponds to 100%. SD, standard deviation; NTR, nothing to report

| **ID** | **1. How satisfied are you in general with your experience with this training program?** | **2. Do you think this application is easy to use?** | **3. Do you think this application is structured in a functional and practical way?** | **4. Were the explanations about using the application clear?** | **5. Are the movements made by the avatar easy to understand?** | **6. Did you feel safe while exercising with the Parkinson Rehab® application?** | **7. Do you think that using the application was a valid tool for doing exercises at home?** | **8. Would you recommend this application to other people with Parkinson's disease?** | **9. What did you like most about your experience using the Parkinson Rehab® application?** | **10. What did you like least about your experience using the Parkinson Rehab® app?** | **Total score** |
| --- | --- | --- | --- | --- | --- | --- | --- | --- | --- | --- | --- |
| 1 | 8 | 6 | 10 | 10 | 7 | 10 | 9 | 7 | User-friendly | Time commitment | 67 (83.75%) |
| 2 | 10 | 8 | 10 | 10 | 10 | 10 | 10 | 10 | Perceived  benefits | NTR | 78 (97.50%) |
| 3 | 9 | 10 | 10 | 10 | 10 | 10 | 9 | 10 | User-friendly | Technological issues | 78 (97.50%) |
| 4 | 10 | 10 | 10 | 8 | 10 | 10 | 10 | 10 | Perceived  benefits | NTR | 78 (97.50%) |
| 5 | 10 | 10 | 10 | 10 | 10 | 10 | 10 | 7 | Perceived  benefits | NTR | 77 (96.25%) |
| 6 | 10 | 8 | 10 | 10 | 7 | 10 | 10 | 5 | Training | Technological issues | 70 (87.50%) |
| 7 | 8 | 8 | 9 | 9 | 10 | 7 | 10 | 9 | Training | Technological issues | 70 (87.50%) |
| 8 | 8 | 8 | 8 | 8 | 8 | 8 | 8 | 8 | NTR | NTR | 64 (80%) |
| 9 | 8 | 8 | 7 | 8 | 8 | 7 | 7 | 7 | Perceived  benefits | NTR | 60 (75%) |
| 10 | 10 | 10 | 10 | 10 | 10 | 10 | 10 | 9 | Training | NTR | 79 (98.75%) |
| 11 | 10 | 10 | 10 | 10 | 10 | 10 | 10 | 10 | User-friendly | NTR | 80 (100%) |
| 12 | 8 | 7 | 7 | 10 | 8 | 8 | 8 | 5 | Training | Time commitment | 61 (76.25%) |
| 13 | 9 | 7 | 8 | 8 | 9 | 9 | 7 | 8 | Perceived  benefits | Technological issues | 65 (81.25%) |
| 14 | 10 | 10 | 9 | 10 | 7 | 10 | 8 | 10 | Training | NTR | 74 (92.50%) |
| 15 | 9 | 10 | 8 | 10 | 10 | 9 | 10 | 9 | User-friendly | NTR | 75 (93.75%) |
| 16 | 8 | 10 | 10 | 9 | 8 | 8 | 6 | 8 | NTR | No perceived  benefits | 67 (83.75%) |
| 17 | 10 | 8 | 10 | 10 | 10 | 10 | 10 | 10 | NTR | NTR | 78 (97.50%) |
| 18 | 6 | 2 | 10 | 10 | 6 | 7 | 8 | 6 | Training | Technological issues | 55 (68.75) |
| **Mean ± SD** | 8.94 ± 1.16 | 8.33 ± 2.06 | 9.22 ± 1.11 | 9.44 ± 0.86 | 8.78 ± 1.40 | 9.06 ± 1.21 | 8.89 ± 1.32 | 8.22 ± 1.73 |  |  | 70.89 ± 7.66 |

**Table 2.** Results of the survey evaluating app usability and general experience with the training protocol. Questions 1-8 were assessed with a 10-point numerical rating scale (0 = "not at all" - 10 = "extremely"). Positive aspects (question 9) were categorized in " Training ", " User-friendly", and " Perceived benefits "; negative aspects (question 10) were classified in "Technological issues", "Time commitment", and " No perceived benefits”.

Column "Total score" reports: (i) the sum of scores obtained from questions 1-8, (ii) a percentage score (in brackets) in which a score of 80 corresponds to 100%. SD, standard deviation; NTR, nothing to report.
