# Supplementary material for: Home-based exercise training by using a smartphone app in patients with Parkinson’s disease: a feasibility study
Source: Front Neurol. 2023 Jun 28;14:1205386. doi: 10.3389/fneur.2023.1205386 (PMC10338039; doi:10.3389/fneur.2023.1205386)
Supplement: Supplementary file 3 [file Table_3.DOCX]

**Description of the exercises included in the training program**

| **Laying** |  |
| --- | --- |
| Exercise 1 | Position: supine.  Movement: extended arm and contralateral flexed leg raise (simultaneous).  Repeat on the opposite side. |
| Exercise 2 | Position: quadrupedal.  Movement: forward arm extension and backward contralateral leg extension.  Repeat on the opposite side. |
| Exercise 3 | Position: supine with hips and knees flexed (feet on the floor) and abducted arms (90°).  Movement: alternated whole-body rotation along the longitudinal axis from side to side. |
| Exercise 4 | Position: supine with hips and knees flexed (feet on the floor).  Movement: hip extension (glutes off the floor). |
| Exercise 5 | Position: supine  Movement: simultaneous maximal hip and knee flexion (i.e., knee to the chest); holding the knee with the ipsilateral arm.  Repeat on the opposite side. |
| Exercise 6 | Position: supine with hips and knees flexed (feet on the floor) and abducted shoulder (90°).  Movement: alternated pelvis rotation along the longitudinal axis from side to side. |
| Exercise 7 | Position: supine, arms along the trunk, flexed elbows and hands facing upwards  Movement: extension of the arms backwards.  Repeat on the opposite side. |
| Exercise 8 | Position: supine.  Movement: lying-to-sitting postural transition.  Repeat on the opposite side. |
| Exercise 9 | Position: long sitting with hands on the floor (back)  Movement: head and trunk extension |
| Exercise 10 | Position: quadrupedal.  Movement: Cat-Cow exercise. |
| Exercise 11 | Position: side-lying.  Movement: extended arm and leg abduction (toward the ceiling) while holding a bottle of water.  Repeat on the opposite side. |
|  |  |
| **Sitting position (without back support)** | |
| Exercise 1 | Position: sitting, flexed shoulders (90°) and arms (full flexion) and closed fists.  Movement: arms upward extension (alternated). |
| Exercise 2 | Position: sitting, shoulders abducted (90°), arms flexed (full flexion) and closed fists.  Movement: bilateral arm extension on frontal plane and simultaneous head rotation to one side (alternated).  Slow movements. |
| Exercise 3 | Position: sitting, holding a stick with both hands (extended arms).  Movement: shoulder flexion/extension (full available range of motion). |
| Exercise 4 | Position: sitting, flexed shoulders (90°), holding a stick with both hands (extended arms).  Movement: side-to-side trunk flexion on the frontal plane. |
| Exercise 5 | Position: sitting, flexed shoulders (90°), holding a stick with both hands (extended arms).  Movement: side-to-side trunk rotation along the longitudinal axis. |
| Exercise 6 | As for ‘Exercise 2’ but with increased speed. |
| Exercise 7 | Position: sitting.  Movement: feet plantarflexion/dorsiflexion (alternated) |
| Exercise 8 | Position: sitting, shoulder abducted (90°) and arms extended  Movement: trunk rotation along the longitudinal axis so to touch the contralateral hand (alternated). |
| Exercise 9 | Position: sitting  Movement: full sit-to-stand transfer and back. |
| Exercise 10 | Position: sitting, soles together  Movement: trunk flexion/extension |
| Exercise 11 | Position: sitting, shoulders abducted (90°), arms flexed (full flexion) and closed fists.  Movement: explosive arms extension coupled with opening hands (alternated). |
| Exercise 12 | Position: sitting  Movement: side-to-side complete trunk rotation associated to an upwards extension of the arm and backwards extension of the ipsilateral leg (alternated). |
| **Standing** |  |
| Exercise 1 | Position: standing,  Movement: trunk rotation (90°) combined with a lateral step on the frontal plane while opening both arms (alternated). |
| Exercise 2 | Position: standing  Movement: simultaneous maximal hip and knee flexion (i.e., knee to the chest) and ipsilateral shoulder flexion (180°).  Repeat on the opposite side. |
| Exercise 3 | Position: standing, feet apart.  Movement: lateral weight shifting with overhead reaching task (alternated). |
| Exercise 4 | Position: standing  Movement: forward lunges with arm  Forwards frontal lunges with simultaneous arm opening. Repeat with the contralateral side. |
| Exercise 5 | Position: split-stance  Movement: antero-posterior weight shifting coupled with arm swing.  Repeat with inverted split-stance. |
| Exercise 6 | Position: standing  Movement: reaching movement from overhead to floor.  Repeat on the opposite side. |
| Exercise 7 | Position: standing.  Movement: lateral weight shifting followed by a forward half lunge, and back Repeat on the opposite side. |
| Exercise 8 | Position: standing.  Movement: forward-backward frontal lunges.  Repeat on the opposite side. |
| Exercise 9 | Position: standing.  Movement: lateral weight shifting followed by a forward long step, and back Repeat on the opposite side. |
| Exercise 10 | Position: standing.  Movement: lateral weight shifting, ipsilateral trunk flexion (frontal plane) and contralateral hip abduction (with extended leg).  Repeat on the opposite side. |
| Exercise 11 | Position: standing.  Movement: trunk rotation (90°) along the longitudinal axis, ipsilateral shoulder abduction (90°) and lateral step (simultaneous).  Alternated with the opposite side. |
| Exercise 12 | Position: standing.  Movement: turning (180°) starting with the ipsilateral leg to the chosen turning direction.  Repeat on the opposite direction. |
| Exercise 13 | Position: standing.  Movement: walking 4 long steps forward and 4 long steps backward. |
| Exercise 14 | Position: standing, shoulders flexed (90°), arms flexed (full flexion) and closed fists.  Movement: upward arm extension (alternated) |
| Exercise 15 | Position: standing.  Movement: turning (360°) starting with the ipsilateral leg to the chosen turning direction.  Repeat on the opposite direction. |
| Exercise 16 | Position: standing.  Movement: slow stepping on the spot. |
| Exercise 17 | Position: standing.  Movement: tandem walking with shoulder abduction (90°) (simultaneous). |
| Exercise 18 | Position: standing (with support).  Movement: simultaneous maximal hip and knee flexion (i.e., knee to the chest; holding the knee with the ipsilateral hand.  Repeat on the opposite side. |
| Exercise 19 | Position: standing.  Movement: walking 2 steps on a straight line followed by obstacle crossing. |
